# Progress in Diagnosis and Treatment of Primary Immunodeficiencies

**DOI:** 10.3390/jcm14186363

**Published:** 2025-09-09

**Authors:** Alexandros Grammatikos

**Affiliations:** Department of Immunology, North Bristol NHS Trust, Bristol BS10 5NB, UK; alexandros.grammatikos@nbt.nhs.uk

Primary immunodeficiencies (PIDs), or elsealso called inborn errors of immunity, are a heterogenous group of genetic disorders characterized by immune system dysfunction. They are usually associated with recurrent, opportunistic, or severe infections, but also various autoimmune and inflammatory manifestations can be seen in these patients. Improved awareness and advances in molecular diagnostics have led to a significant increase in the number of PIDs that are currently being recognized. Despite a significant expansion in our knowledge in recent years, there remain significant gaps in our understanding of their pathogenesis and potential treatments. In this special issue recent developments and future prospects on the diagnosis and management of this diverse group of disorders are discussed.

Common Variable Immunodeficiency (CVID) is one of the most common PIDs, and typically patients suffer from recurrent bacterial sinopulmonary infections. Viral infections are also becoming increasingly recognized to be an issue and Al-Hakim et al. [1] discuss their impact in CVID and CVID-like disorders. Higher morbidity and mortality from COVID-19 infection were observed during the recent pandemic, although vaccination and antiviral therapies have since improved outcomes. Other respiratory infections like rhinovirus, RSV, and influenza are also more common. Severe mucocutaneous infections from HPV, HSV, and VZV have all been reported and are often linked to T-cell dysfunction or specific genetic mutations. Gastrointestinal infections by norovirus, CMV, and HSV can all cause significant morbidity and mortality in these patients. Viral meningoencephalitis, often caused by enteroviruses, is another rare but severe complication. Overall, the authors highlight the need to remain vigilant for these infections. Levels of circulating T- [1] and B-cells [2] can be used to stratify patients, with lower levels being associated with a higher risk of infections. Immunoglobulin replacement therapy remains the cornerstone for prevention, but for treatment, much research is still required to develop targeted therapies for a wider array of pathogens.

Zinser et al. [3], on the other hand, review the various hepatic complications that can arise in PID. As expected, infections are more common, with various viral (e.g., CMV, EBV), bacterial (e.g., *Staphylococcus aureus*), mycobacterial, parasitic (e.g., *Cryptosporidium*), and fungal (e.g., *Aspergillus*) infections being reported. The authors emphasize the need for a more cautious diagnostic approach in these patients, as serology investigations are often unreliable. Non-infective complications are also becoming increasingly recognized and contribute to significant morbidity and mortality. Nodular regenerative hyperplasia, often associated with non-cirrhotic portal hypertension, is of particular concern in CVID. No evidence-based treatments are currently available for this, and early diagnosis is vital so that potentially reversible causes can be managed. Autoimmune hepatitis, granulomas, primary biliary cholangitis, and primary sclerosing cholangitis are potential autoimmune complications that arise. Immunosuppressive drugs are the hallmark of their treatment, but a fine balance needs to be kept in view of the heightened risk of infection. Iatrogenic liver injury is also becoming increasingly common as the spectrum of therapeutic interventions for PID is expanding. These patients often require prolonged courses or higher doses of antimicrobials, which can sometimes cause liver injury. Although less common, hepatic malignancies are also seen, with non-Hodgkin’s lymphoma being of particular concern. Blood tests (e.g., full blood count, liver function tests, clotting profile, various infectious screens), imaging studies (e.g., ultrasonography, computed tomography scan, or magnetic resonance imaging), and liver biopsies are all helpful in detecting these complications at an early stage [4].

Syndromic primary immunodeficiencies (sPIDs) are conditions with features of PID overlapping with other multisystem clinical manifestations, which are not directly linked to the immunologic deficit. They are caused by single-gene disorders, metabolic aberrations, or chromosomal abnormalities. Ng et al. [5] present data from a retrospective study in children followed up at the Bristol Royal Hospital for Children, in the UK. Thirty-six children with sPIDs were identified, with the most common being DiGeorge (*n* = 5), ataxia telangiectasia (*n* = 4), STAT3 deficiency (*n* = 4), Wiskott–Aldrich (*n* = 4), Kabuki (*n* = 3), and sideroblastic anemia with B-cell immunodeficiency (*n* = 3) syndrome. The majority of patients suffered from recurrent and/or severe infections (92%), with the most common extra-immunological manifestations being dysmorphism (72%) and disorders of the nervous system (78%). The authors emphasize the need to keep a low threshold to clinically assess and investigate patients with syndromic features for these disorders. Severe, opportunistic, or frequent infections should all raise suspicion, but immune dysregulation, autoinflammation, and lymphoproliferation can also be presenting features. Immunological investigations (serum immunoglobulins, vaccine responses, lymphocyte subsets, etc.), radiosensitivity assays, karyotyping, and molecular genetic testing are all helpful in diagnosing these conditions [6].

Costagliola et al. [7] focus on DiGeorge syndrome, also known as 22q11.2 deletion syndrome (22q11.2 DS). In these patients the thymus is often hypoplastic or absent, leading to various immune system abnormalities. 22q11.2 DS patients have an extremely variable clinical phenotype, with some presenting with recurrent or severe infections, while others have immune dysregulation, atopic diseases, or extra-immunological manifestations. The authors divided 34 patients into three groups and assessed their immunophenotypic characteristics. Patients with increased or severe infections exhibited a significant reduction in their CD3+ and CD4+ cells, which likely explains this predisposition. Patients with an immune dysregulation phenotype showed skewing towards increased memory T-cell populations, with reduced levels of B-cells and recent thymic emigrants (RTEs), suggesting a more profound thymic impairment. In contrast, patients without infective complications or immune dysregulation exhibited the highest levels of RTEs. This study complements previous efforts in this field [8] and offers a valuable tool in identifying patients that are at risk of infections or immune dysregulation.

Finally, Sikhayeva et al. [9] report on the clinical characteristics of PID patients from the Republic of Kazakhstan. Patient registries worldwide provide a valuable source of information to study the epidemiological characteristics, natural disease course, diagnosis, treatment, and survival of PIDs [10,11]. Two hundred and sixty-nine patients diagnosed with PID were identified in the period between 2009 and 2023: 139 males and 130 females. The average detection rate was 14 new cases per year, with a mean age at diagnosis of 11.3 years. The predominant clinical manifestations were severe or recurrent infections (66%), growth retardation (47%), and autoimmune manifestations (16%). Severe infections led to hospitalization in 13.3% of cases, with pneumonia and sepsis being most common. Humoral immunodeficiencies were the most common diagnosis (*n* = 120, 44.6%), with the majority of cases being CVID. Complement deficiencies were seen in 83 patients (30.8%), all due to C1 inhibitor deficiency. Combined immunodeficiencies were seen in 35 (13%), with severe combined immunodeficiency (SCID) having a particularly high (50%) mortality rate. Finally, phagocytic cell defects were seen in 12 cases (4.5%). A total of 169 patients (62.8%) underwent genetic testing, and pathogenic variants were detected in BTK, DOCK8, STAT3, WAS, ATM, AIRE, FAS, TBX1, and other genes. The authors conclude that there is an urgent need for better diagnosis, neonatal screening, and improved access to treatments for these patients in the Republic of Kazakhstan. 

The above studies offer an intriguing glimpse into the rapidly evolving field of PID. Innovations like next-generation sequencing, improved newborn screening, gene therapy, targeted biologics, and hematopoietic stem cell transplantation offer significant hope for these patients. However, important challenges remain to be tackled, with equitable access to these advances globally being of utmost priority. As the field evolves, multidisciplinary collaboration and sustained investment in research will be essential to realize the full potential of these breakthroughs and ensure that every PID patient receives timely, precise, and effective care.
